# Hashimoto Thyroiditis and Dyslipidemia in Childhood: A Review

**DOI:** 10.3389/fendo.2019.00868

**Published:** 2019-12-10

**Authors:** Rade Vukovic, Aleksandra Zeljkovic, Biljana Bufan, Vesna Spasojevic-Kalimanovska, Tatjana Milenkovic, Jelena Vekic

**Affiliations:** ^1^Department of Pediatric Endocrinology, Mother and Child Healthcare Institute of Serbia “Dr Vukan Cupic”, Belgrade, Serbia; ^2^School of Medicine, University of Belgrade, Belgrade, Serbia; ^3^Department of Medical Biochemistry, Faculty of Pharmacy, University of Belgrade, Belgrade, Serbia; ^4^Department of Microbiology and Immunology, Faculty of Pharmacy, University of Belgrade, Belgrade, Serbia

**Keywords:** Hashimoto autoimmune thyroiditis, children, dyslipidemia, novel lipid biomarkers, L-thyroxine treatment

## Abstract

Hashimoto autoimmune thyroiditis (AIT) is the most common cause of acquired hypothyroidism in the pediatric population. Development of AIT is mediated mainly by cellular immune response directed toward thyroid autoantigens, leading to inflammation and impaired function of thyroid gland. Both thyroid dysfunction and inflammation affect the metabolism of plasma lipoproteins. The alterations in lipid profile worsen with the advancement of hypothyroidism, ranging from discrete changes in euthyroid AIT patients, to atherogenic dyslipidemia in the overt hypothyroidism. In this review, characteristics of dyslipidemia in pediatric AIT patients, and the consequences in respect to the risk for cardiovascular disease (CVD) development are discussed. Additionally, benefit of L-thyroxine treatment on serum lipid profile in pediatric AIT patients is addressed. Finally, potential usefulness of novel lipid biomarkers, such as proprotein convertase subtilisin/kexin type 9 (PCSK9), non-cholesterol sterols, low-density lipoprotein particle size and number, and high-density lipoprotein structure and functionality in AIT patients is also covered. Further longitudinal studies are needed in order to elucidate the long-term cardiovascular outcomes of dyslipidemia in pediatric patients with Hashimoto AIT.

## Introduction

Hashimoto autoimmune thyroiditis (AIT) is the most common cause of acquired hypothyroidism in childhood and adolescence. The prevalence of AIT in childhood peaks at early to mid- puberty. Presentation of the disease is rare before the age of 3 years, but there are described cases in infancy, too (1). Female strong preponderance has been reported with female to male ratio up to 3.4:1 (1–3), with high prevalence in patients with Down and Turner syndrome (4). Clinical manifestations of AIT in childhood are extremely diverse, ranging from completely normal, asymptomatic state, to pronounced symptoms of severe thyroid dysfunction.

Thyroid hormones have a broad spectrum of physiological effects on lipoprotein metabolism. As a result, plasma lipid and lipoprotein levels are sensitive to changes in the thyroid hormones concentrations. The alterations in lipid profile accompanying AIT worsen along with the advancement of hypothyroidism, ranging from discrete pro-atherogenic markers in euthyroid AIT, to full-blown dyslipidemia in many patients with the overt hypothyroidism (5–7). Furthermore, autoimmune disease itself has significant impact on lipid profile, as evidenced by a high prevalence of dyslipidemia in patients with autoimmune diseases (8–10), which may account, at least in part, to the increased cardiovascular disease (CVD) risk. Thus, it could be regarded as convenient that the efficacy of L-thyroxine (L-T4) treatment in the normalization of lipid status is directly proportionate to the degree of thyroid dysfunction, being highest in the overt hypothyroidism (5, 7, 11, 12). However, the waist majority of data linking autoimmune thyroid disease with dyslipidemia were gained from the studies in adults (13), whereas data in pediatric populations are limited. Also, data is scarce regarding the effects of L-T4 treatment on lipid profile in pediatric hypothyroidism, with or without thyroid autoimmunity (14–18).

In this narrative review, we will discuss recent findings regarding the effects of AIT on lipid metabolism and CVD risk, including the impact of L-T4 treatment on dyslipidemia and potential use of novel lipid biomarkers in pediatric patients with AIT.

## Development and Clinical Manifestations of Hashimoto's Thyroiditis in Childhood

Like other autoimmune diseases, AIT is multifactorial disease caused by complex interplay of genetic (1, 19–26), environmental (21, 27–29), and hormonal factors (19, 21, 30), that provoke the inappropriate immune response against thyroid gland. HT is mainly mediated by cellular immune response directed toward thyroid autoantigens, leading to inflammation, fibrosis, and impaired function of thyroid gland (4, 26). The first step in pathogenesis is believed to be activation of autoreactive CD4+ T cells i.e., T helper (Th) cells specific for thyroid autoantigens. Th cells type 1 (Th1) activate cytotoxic T lymphocytes (CD8+ lymphocytes) and macrophages, which directly destroy thyroid follicular cells (31). Another subset of Th cells with a role in development and progress of chronic inflammation and tissue damage in HT are Th17 cells. Higher proportion of Th17 cells, as well as higher levels of cytokines produced by these cells were found in peripheral blood and thyroid tissue in HT patients compared with healthy controls (32–34). It is also observed that T regulatory (Treg) cells, cells with immunosuppressive function, accumulate in thyroid tissue of HT patients. However, in these patients Treg cells were found to be dysfunctional (35, 36). B lymphocytes, although representing humoral immunity, are also activated in AIT, producing antibodies against thyroid autoantigens (26). These cells are part of thyroid lymphocyte infiltrate (37) and exert antibody synthesis in the gland (31, 38). Autoantibodies are crucial component in AIT pathogenesis, since antibody-dependent cell-mediated cytotoxicity is another and important factor responsible for apoptosis of thyroid follicular cells in this disease (26, 31).

Clinical presentation of AIT is best reviewed with respect to the thyroid status, since children with AIT can present as completely euthyroid, with mild subclinical hypothyroidism, severe overt hypothyroidism, or in the state of subclinical or overt hyperthyroidism (Hashitoxicosis) (39–43). Majority of children with AIT are either euthyroid or subclinically hypothyroid at the time of diagnosis (41, 42). Euthyroid state, defined by thyroid function tests within normal range, is usually asymptomatic, besides the frequent finding of a goiter (41, 42, 44, 45). Subclinical hypothyroidism in AIT, defined by elevated TSH with normal levels of serum thyroid hormones (fT4 and fT3), is usually classified as mild (TSH 4.5–10 mIU/l) or severe (TSH > 10 mIU/l) (7, 41, 46–49). Although the very name—“subclinical hypothyroidism” implies that this form of thyroid dysfunction presents merely as a laboratory finding without any signs or symptoms of clinical hypothyroidism besides goiter, these patients actually may present with other clinical and laboratory findings (7, 45, 47, 50, 51). Typical clinical signs and symptoms of hypothyroidism have been reported in some children with subclinical hypothyroidism, as well as the improvement of hypothyroidism symptoms scores with L-T4 treatment (7, 47, 51). Also, untreated long-lasting subclinical hypothyroidism in children has been firmly associated with subtle pro-atherogenic alterations in lipid profile (7, 17, 52). On the other hand, currently available data indicate that children with untreated longstanding subclinical hypothyroidism have normal linear growth, neurocognitive and behavioral outcomes, and bone health status (7, 47, 48, 53). It should be noted that although the association of obesity and subclinical hypothyroidism is well-documented, abnormal thyroid function in obese patients seems to be a consequence of obesity, rather than a cause (47, 48).

Overt hypothyroidism, defined by elevated TSH with low level of serum fT4, is present in ~20% of all children with AIT at the time of diagnosis, and the onset of clinical manifestations is usually subtle (41, 42, 46). Classical signs and symptoms of overt hypothyroidism which may be seen in these children are: goiter, constipation, weight gain, poor growth velocity or short stature, fatigue and somnolence, poor school performance, cold intolerance, dry skin, bradycardia, yellowish-pale skin tone with facial puffiness (myxedema), with frequent laboratory findings of anemia and dyslipidemia (41, 42, 46). Adolescents with overt hypothyroidism can also present with delayed or arrested pubertal development, irregular menstrual periods, menometrorrhagia, or amenorrhea in girls (41, 46). Rarely, girls with longstanding severe overt hypothyroidism can present with precocious puberty and menstrual bleeding with hyperprolactinaemia and delayed bone age (Van Wyk-Grumbach syndrome) (42, 46, 54).

Hashitoxicosis, the initial hyperthyroid phase of AIT caused by the release of preformed thyroid hormones from the gland, can be detected in ~10%, and subclinical hypothyroidism in up to 3% of children with AIT (39–43, 55–57). Clinical signs and symptoms of children with hashitoxicosis are those of hyperthyroidism, and cannot be distinguishable can be indistinguishable from Grave's disease: goiter, tachycardia, tremor, weight loss, restlessness, warm moist skin, ophthalmopathy, growth acceleration, delayed, or precocious puberty (39, 41, 58). Fortunately, this hyperthyroid phase of AIT is transient, usually resolving within several months into euthyroid state, or progressing to permanent hypothyroidism (41, 55, 56).

Apart from the described symptoms caused by the thyroid dysfunction itself, children with AIT may also have other autoimmune diseases or syndromes, such as celiac disease, type 1 diabetes (T1DM), Down's or Turner's syndrome, with corresponding symptoms adding to the overall clinical picture (42, 46).

## Hashimoto Thyroiditis and Dyslipidemia

**Figure 1:**
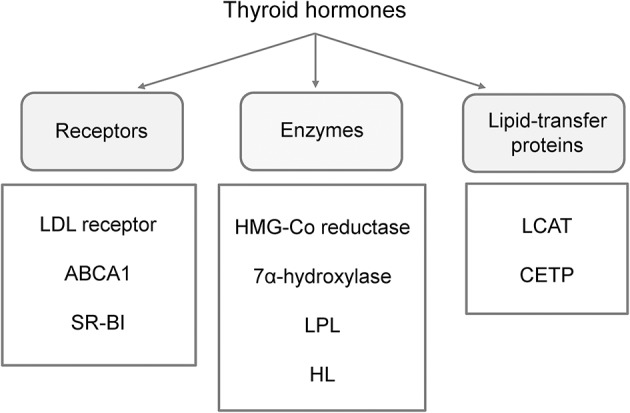
Main effector molecules involved in alterations of lipoprotein metabolism driven by thyroid hormones. LDL, low-density lipoprotein; ABCA1, ATP binding cassette subfamily A member 1; SR-BI, scavenger receptor class B type 1; LPL, lipoprotein-lipase; HL, hepatic lipase; LCAT, lecithin:cholesterol acyltransferase; CETP, cholesteryl-ester transfer protein.

In general, subclinical hypothyroidism can slow-down metabolic pathways of cholesterol uptake, synthesis, and secretion, as well as reverse cholesterol transport process and catabolism of triglyceride (TG)-rich lipoproteins (Figure 1) (12, 59, 60). As compared to healthy children, total and low-density lipoprotein cholesterol (LDL-C) levels are commonly elevated, while the level of high-density lipoprotein cholesterol (HDL-C) can be normal or decreased in patients with subclinical hypothyroidism (Table 1). Although some authors reported no differences in serum lipid profile between pediatric patients with subclinical hypothyroidism and controls, the frequency of dyslipidemic children was significantly higher in the patients group (51, 61). The study evaluating children, adolescents and adults with subclinical hypothyroidism suggested that abnormalities in lipid profile are more pronounced in adult patients, as well as in those with severe form of the disease (62). Yet, the impact of the disease severity on lipid profile was not confirmed in later studies in pediatric patients (61, 63).

**Table 1:** Lipid profile of pediatric patients with subclinical hypothyroidism.

**Authors**	***N***	**Age, years**	**Alterations of lipid profile**	**References**
Paoli-Valeri et al. (2005)	17	4.3 ± 1.0	↓HDL-C; ↔TC; ↔ LDL-C; ↔ TG; ↔ TC/HDL-C; ↔ LDL-C/HDL-C	(14)
Cerbone et al. (2014)	49	8.5 ± 0.5	↓HDL-C; ↑TC/HDL-C; ↑TG/HDL-C; ↔TC; ↔ LDL-C; ↔ TG; ↔ non-HDL-C	(17)
Dahl et al. (2018)	228	13.3 ± 4.2	↑TC; ↑non-HDL-C; ↔ HDL-C	(18)
Catli et al. (2014)	27	10 (6.9)^*^	↔TC; ↔LDL-C; ↔HDL-C; ↔TG	(51)
Cerbone et al. (2016)	39	9.2 ± 3.6	↓HDL-C; ↑TC-/HDL-C; ↑TG/HDL-C; ↔TC; ↔ TG; ↔ LDL-C; ↔ non-HDL-C	(52)
Unal et al. (2017)	38	8.1 ± 3.6	↑TC; ↑LDL-C; ↑LDL-C/HDL-C; ↑TC/HDL-C; ↔ HDL-C; ↔ TG	(61)
Marwaha et al. (2011)	280/35^#^	12.8 ± 2.8	↔/↓HDL-C; ↔/↔TC; ↔/↑ TG; ↔/↑ LDL-C	(62)
Isguven et al. (2016)	66	14.4 ± 2.4	↑TC; ↑LDL-C; ↔ TG; ↔ HDL-C	(63)

*In relation to euthyroid or control group: ↑, increased; ↓, reduced; ↔, unchanged*.

* *Median (interquartile range) was reported*.

# *280 patients with TSH ≤ 10 mIU/L and 35 patients with TSH > 10 mIU/L were studied. TC, total cholesterol; LDL-C, low-density lipoprotein cholesterol; HDL-C, high-density lipoprotein cholesterol; TG, triglycerides; non-HDL-C, non-high-density lipoprotein cholesterol*.

The hallmark of subclinical hypothyroidism-related dyslipidemia is reduced synthesis of liver LDL receptors and the mechanisms behind this effect have been extensively studied and explained. Emerging evidence suggests that the levels of circulating proprotein convertase subtilisin/kexin type 9 (PCSK9), a serin-protease responsible for downregulation of liver LDL receptors, is also increased in subclinical hypothyroidism (64), paving the way for innovative lipid-lowering therapy in this category of patients (5). However, data on PCSK9 in children are sparse and the studies examining efficacy, safety, and tolerability of PCSK9 inhibitors in pediatric patients are underway (65).

Body cholesterol pool is maintained by delicate balance between the processes of cholesterol synthesis, absorption, and biliary secretion, all of which can be affected even by subtle alterations in thyroid hormones levels (60). Plasma non-cholesterol sterols, including cholesterol precursors and plant sterols, are validated biomarkers of cholesterol biosynthesis and intestinal absorption efficiency (66). The results of a small study by Matysik et al. (67) suggested that the levels of plasma non-cholesterol sterols could serve as indicators of disrupted cholesterol homeostasis in patients with hyper- and hypothyroidism. Recently, plasma profile of non-cholesterol sterols from birth to 15 years of age was characterized in pediatric population without dyslipidemia (68). These data could form a solid base for future evaluation of the extent of cholesterol synthesis and absorption alterations in children and adolescents with AIT.

The lack of thyroid hormones is associated with reduced clearance of TG-rich particles, due to attenuated lipoprotein-lipase (LPL) and hepatic lipase (HL) activities, and increased production of very-low density lipoprotein (VLDL) particles (5). Hence, apart from the impact on LDL-C level, thyroid dysfunction may affect qualitative characteristics and functional properties of LDL particles. Namely, hypertriglyceridemia is intimately linked to the increased production of small, dense LDL particles (69). Bearing in mind that thyroid hormones may protect LDL particles from oxidation (70), and the fact that small, dense LDL particles are prone to oxidative modifications (69), there is increased potential for adverse modification of LDL particles in hypothyroid state. Indeed, increased small, dense LDL, and oxidized LDL particles were recently reported in normolipidemic adult patients with hypothyroidism (71). These data clearly demonstrated the usefulness of advanced lipid testing for identification of the patients with high CVD risk, which should be further confirmed in pediatric patients with both subclinical and overt thyroid dysfunction.

In contrast to firm scientific and clinical evidence which consistently points to elevated LDL-C concentrations in patients with hypothyroidism, data regarding HDL-C are not homogenous. As it has been presented in Table 1, HDL-C levels were decreased or unchanged in hypothyroid states. Interpretation of data and drawing of conclusions is particularly complicated in pediatric population, since most of the data regarding the association of HDL and thyroid status is derived from studies in adults. It is also noteworthy that studies analyzing HDL-C concentration in children with hypothyroidism were conducted in smaller cohorts, which might affect the reliability of the obtained results. Therefore, larger studies with prospective design are needed to resolve this issue.

**Figure 2:**
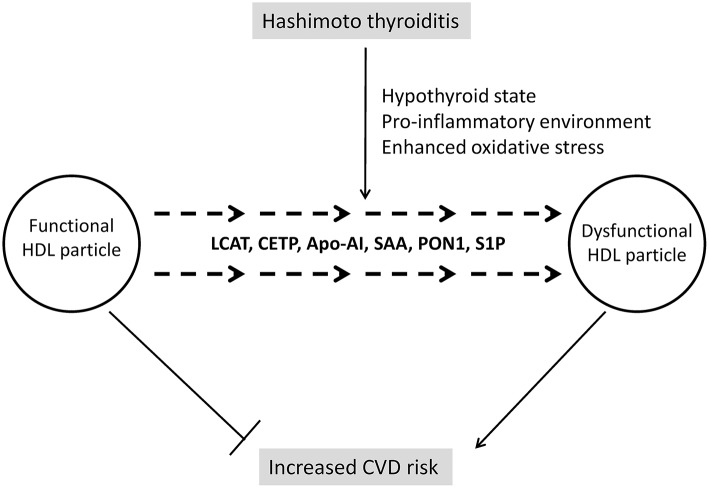
Changes of HDL structure and functionality in Hashimoto thyroiditis. LCAT, lecithin:cholesterol acyltransferase; Apo-AI, apolipoprotein A-I; SAA, serum amyloid A; PON1, paraoxonase 1; S1P, sphingosine-1-phosphate.

However, a contemporary approach to HDL's clinical significance might put aside these conflicting results concerning HDL-C levels in hypothyroid subjects, since another question is considered as even more important in modern research and clinical practice. Namely, due to complex structure and numerous functions of HDL, it is nowadays accepted that quality of this lipoprotein's particles is more significant than their cholesterol content (72). As precisely summarized in a review by Triolo et al. (73), functional properties of HDL could be grouped in four essential categories: reverse cholesterol transport, antioxidative, anti-inflammatory, and vasodilatatory activities. It has also been demonstrated that alterations in HDL structure affect its functionality. In addition, it is now clear that both HDL structure and functions can be easily modified if changes occur in their vascular environment (74). Therefore, several new aspects should be considered regarding HDL in patients with AIT, including the impact of subclinical or overt hypothyroidism on HDL's quality, as well as the influence of pro-inflammatory and pro-oxidative environment on these particles. Providing answers on these questions might have significant implications for interpretation of CVD risk in AIT patients, especially those of young age, considering their long-term exposure to possible detrimental factors (Figure 2).

Changes of HDL's structure and function in relation to thyroid hormones status are largely unexplored. However, a recent study has shown that both cholesterol efflux and activity of HDL-associated enzyme paraoxonase 1 (PON1) are decreased in patients with overt hypothyroidism (75), thus implicating diminishing of HDL functionality. Several reasons could be responsible for such findings. Namely, studies involving human subjects and animal models have demonstrated that hypothyroidism is associated with decreased activities of cholesteryl-ester transfer protein (CETP), lecithin:cholesterol acyltransferase (LCAT), and HL (76, 77). Having in mind that these enzymes are key regulators of HDL metabolism, changes in HDL structure and consequently function should be expected. Indeed, a higher prevalence of larger HDL 2 subclasses was found in subjects with hypothyroidism in a study by Tan et al. (76). Larger HDL particles are generally considered as highly atheroprotective, but, as it has been already mentioned, novel data (75) suggest that functionality of HDL particles in hypothyroidism is compromised.

The next aspect that should be considered is the impact of inflammation on HDL particles. So far, changes in both HDL-C level and HDL structure have been reported in several autoimmune diseases (78). It is also noteworthy that AIT is the most frequent co-morbidity of pediatric patients with T1DM (79). In our recent study, we analyzed lipid and lipoprotein subclasses profile in pediatric T1DM patients with and without co-existing autoimmune diseases and found that those with associated Hashimoto AIT had more profound dyslipidemia (80). Generally speaking, HDL possesses strong anti-inflammatory properties, but it has been demonstrated that pro-inflammatory environment can diminish its protective capacity (78). Being an autoimmune disease, Hashimoto thyroiditis is characterized by chronic inflammation (81). Many components of inflammation are shown to affect HDL particles. Previous researches pointed toward serum amyloid A (SAA), which is abundantly produced in inflammatory states and is capable to replace apolipoprotein AI (apo-AI) on HDL particles, thereby diminishing their anti-inflammatory properties and, paradoxically, turning them into inflammatory agents (78). Moreover, it has been shown that activity of LCAT is reduced (82) as a consequence of inflammation and this can compromise maturation and normal function of HDL particles. In addition, a decrease in CETP mass and activity was also observed in pro-inflammatory conditions (82, 83), although, it was suggested that this could be an adaptive mechanism aimed to prevent massive HDL-C reduction, which is driven by other factors (83). It should also be mentioned that PON1 levels and activity are decreased during inflammation, thereby diminishing antioxidative properties of HDL (84). As for Hashimoto AIT, it has been shown that PON1 level is decreased in these patients (85, 86).

Finally, HDL is a major carrier of sphingosine-1-phosphate (S1P) and evidence suggests that the interaction of S1P with HDL has significant impact on S1P activity (87, 88). S1P is well-known mediator of immune response (89) and recently it has been demonstrated that S1P participates in the development of Hashimoto AIT through its interaction with S1P receptor 1 (90). Yet, whether structural changes of HDL participate in modification of S1P activity in AIT is still to be revealed. Furthermore, the impact of these interactions on increase of CVD risk in Hashimoto AIT, especially in pediatric population, needs to be evaluated.

Before making any conclusions regarding lipid status in AIT, one should be aware that autoimmune disorders frequently aggregate in the same patient. A recent review (91) has shown that various autoimmune comorbidities are associated with AIT in an age-dependent manner. Namely, studies reported that frequency of co-existing autoimmune diseases in children with AIT ranges between 6.6 and 58.2%, wherein celiac disease and T1DM are the most frequent comorbidities (91). Thus, possible contribution of other co-existing autoimmune diseases on lipid profile in AIT should not be neglected.

It is known that T1DM is associated with alterations of serum lipid profile. Semova et al. (92) recently demonstrated decreased cholesterol synthesis and increased cholesterol absorption, with concomitant changes in TC, LDL-C, and HDL-C levels in young patients with T1DM, when compared to age-matched healthy individuals. Moreover, it has been suggested that both structural and functional alterations affect HDL particles in T1DM (93). Similarly, it has been shown that patients with celiac disease exhibit changes in lipid profile, especially decreased HDL-C concentration (94). Yet, it is noteworthy that independent effects of co-existing autoimmune diseases on serum lipids are rarely evaluated. However, it has been reported that LDL-C and TG levels are higher in children with concomitant presence of T1DM and celiac disease, when compared to children with T1DM alone (95). Moreover, lower HDL-C levels were found in children with T1DM if celiac disease was co-existing (96). Also, our own results demonstrated that the prevalence of dyslipidemia was higher in pediatric T1DM patients if celiac disease or AIT were concomitantly present (80). Summarizing all mentioned findings, eventual co-existence of other autoimmune diseases should be taken into account for comprehensive evaluation of dyslipidemia in AIT. Further studies are needed to fulfill a gap in current understanding of mechanisms by which polyautoimmunity is involved in development of lipid disorders.

## Effects of L-thyroxine Treatment on Lipid Status in Hashimoto's AIT

Most of the known data regarding the beneficial effects of L-T4 treatment on lipid profile is derived from studies in adults with overt or subclinical hypothyroidism (12, 75, 97–102). Among the recent studies, Minarikova et al. demonstrated significant improvements of total cholesterol (TC), LDL-C, TG, apoB, atherogenic index of plasma, and LDL subclasses following L-T4 substitution treatment in 40 newly-diagnosed overt hypothyroidism patients with AIT (103). Uniquely designed study of 27 adult patients who underwent total thyroidectomy and radioactive iodine treatment for differentiated thyroid carcinoma, revealed that dynamic changes in thyroid function are associated with corresponding dynamic changes of the lipid profile and HDL function (75). Compared to the baseline levels (on L-T4 treatment), when patients entered the overt hypothyroid state (TSH > 30 mU/L after 4 weeks of L-T4 withdrawal), the levels of TC, TG, LDL-C, apoA-I, and apoB significantly increased, and then again recovered to the baseline levels after 3 months, following the reinstitution of L-T4 treatment. The levels of HDL-C increased during the overt hypothyroid state, with impairment of function (evaluated by cholesterol efflux capacity and PON1 activity), and these changes persisted despite restoration of thyroid hormone levels. It could not be concluded if impaired HDL-C function would also improve after a longer period of follow-up (75).

Results from studies in the adult population also indicate that L-T4 treatment could result in lowering of TPO-Ab levels in patients with AIT (104, 105). This could also potentially lead to the improvement of the lipid profile, having in mind that thyroid autoimmunity with higher TPO-Ab levels is associated with an unfavorable lipid profile irrespective of thyroid function, in both children and adults (13, 63, 80, 106, 107).

Subclinical hypothyroidism in children, with a prevalence of 2–9%, is generally considered a benign condition with a significant chance of remission, however, findings of subtle pro-atherogenic abnormalities in these children highlights the lack of consensus regarding treatment criteria (7, 17, 49, 52, 53, 108–110). Treatment with L-T4, which was usually recommended only in children with goiter, hypothyroidism symptoms or TSH levels > 10 mU/L, is now being recommended by some experts for treatment of mild subclinical hypothyroidism in cases of: positive TPO-Ab, concomitant celiac disease, TSH > 8 mU/L in two repeated measurements, gradually increasing TSH levels, hyperlipidemia and younger patient age (7, 47, 49, 52, 53, 109).

Although the association of thyroid function with atherogenic alterations in lipid profile has been well-documented in children, data is scarce regarding the effects of L-T4 treatment in pediatric hypothyroidism, with or without thyroid autoimmunity (14–18). Dorr et al. observed a decrease of thyroid volume in 25 euthyroid children with Hashimoto's thyroiditis with L-T4 treatment, while changes in lipids were not evaluated (44). Another study also showed reduction of goiter with L-T4 treatment especially in cases of overt hypothyroid, but also in SH and euthyroid children with AIT (45).

Among the very few studies evaluating the effects of L-T4 treatment in children with subclinical hypothyroidism, only two studies investigated changes in the lipid profile (51, 52, 108, 111, 112). In a prospective study by Catli et al., 27 children (median age of 10 years) with subclinical hypothyroidism were treated with L-T4 until achievement of euthyroid state plus 6 months and then compared with euthyroid healthy control group. Seven (26%) of these patients with SH had AIT. Although improvement in the hypothyroidism symptoms score was associated with L-T4 treatment, no significant differences were observed regarding TC, TG, HDL-C, and LDL-C (51). The absence of significant findings could be attributed to the relatively short duration of follow-up as well as low number of patients enrolled. On the other hand, a prospective case-control study by Cerbone et al. discovered significant effects of 2 years of L-T4 treatment on pro-atherogenic markers in children with mild (TSH 4.5–10.0 mU/L) idiopathic subclinical hypothyroidism (52). A total of 39 children (mean age 9.2 years) with mild idiopathic subclinical hypothyroidism were compared with healthy controls. However, in this study patients with detectable TPO-Ab, Tg-Ab, or abnormal thyroid echogenicity on ultrasound were excluded from the study. Mean HDL-C levels were lower, with higher TG/HDL-C ratio and atherogenic index (TC/HDL-C), in subclinical hypothyroidism subjects compared with controls. After 2 years of L-T4 treatment, these parameters improved significantly in the SH group, so no more significant differences between subclinical hypothyroidism subjects and controls were observed. It was concluded that 2 years of L-T4 treatment resulted in an improvement of many lipid abnormalities in children with mild idiopathic subclinical hypothyroidism (52). However, having in mind that patients with AIT were excluded from this study, results should be interpreted with caution in the context of pediatric hypothyroidism caused by AIT. Further studies are needed to evaluate the metabolic effects of L-T4 treatment in hypothyroid children with AIT, compared with treated non-autoimmune hypothyroid children and not treated healthy controls.

## Implications for Cardiovascular Prevention and Future Directions

**Figure 3:**
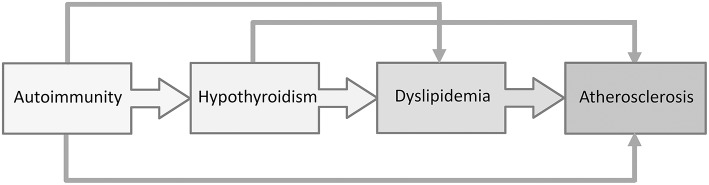
The interplay between Hashimoto thyroiditis, dyslipidemia, and atherosclerosis.

Dyslipidemia and increased carotid intima media thickness (cIMT), a reliable indicator of subclinical atherosclerosis, was recently documented in pediatric patients with Hashimoto thyroiditis (61, 63), suggesting the importance of regular evaluation of cardiovascular risk factors in children with AIT (Figure 3). In recent years, the role of cholesterol in the development of CVD has been challenged. In particular, the main controversy was centered on the impact of dietary fats on cardiovascular risk. According to a recent meta-analysis by de Souza et al. (113), the intake of saturated fatty acids was not associated with a higher cardiovascular risk. Similarly, data from randomized controlled trials shows that the replacement of saturated with polyunsaturated fatty acids might reduce serum cholesterol levels, but does not decrease the risk from all-cause or mortality due to coronary heart disease (114, 115). Nevertheless, a panel of experts from the European Atherosclerosis Society recently issued a Consensus Statement, supported by the evidence from genetic, prospective epidemiologic studies, Mendelian randomization studies and randomized trials evaluating lipid-lowering therapies, that high LDL-C is a causal factor in the pathophysiology of CVD and should remain the main therapeutic target (116).

Although LDL-C level remains primary target for CVD prevention, advanced lipid testing could be advised to reveal hidden cardiovascular risk. In this context, lipoprotein particle size, number and subclasses distribution, HDL lipidome, proteome, and functionality testing may provide valuable information beyond LDL and HDL cholesterol levels. Regarding alterations in metabolism of TG-rich lipoproteins, it can be easily assessed by calculation of non-HDL-C level, as it was recently recommended by expert lipidologists (117). To date, increased PCSK9 levels and its impact on dyslipidema has been reported in the most common autoimmune disease in childhood, T1DM (118), and future studies in pediatric Hashimoto thyroiditis are warranted.

Another important aspect in addressing the link between subclinical hypothyroidism and dyslipidemia is related to the laboratory assessment and interpretation of TSH levels. As stated previously, the definition of subclinical hypothyroidism relies on a mildly elevated serum TSH concentration, which is associated with normal T4 or fT4 levels. According to the guidelines of European Thyroid Association for the management of subclinical hypothyroidism in children, appropriate diagnosis of subclinical hypothyroidism in pediatric patients requires adequate age-adjusted reference ranges for TSH and thyroid hormones is mandatory (119). To date, numerous studies have been performed to define reference range of thyroid hormones in pediatric population, and some of them stratified results according to sex (120–122). Yet, there is still no consensus regarding this issue. Onsesveren et al. (123) recently performed a systematic review of published reference ranges for TSH and fT4 in children and demonstrated substantial differences among studies. For instance, the upper reference limit of TSH ranged between 2.36 and 6.57 mU/L (123). The difference among published reference values could be a consequence of age, gender, and demographic characteristics of included populations, including lifestyle, iodine, and selenium status (120) and/or different assays employed for the laboratory measurements of thyroid hormones. As previously acknowledged by the National Academy of Clinical Biochemistry, serum TSH level has high biological variability, due to short half-life and diurnal variation (124). Also, as a consequence of improved sensitivity and specificity of the methods for TSH determination, the upper reference limit for TSH decreased over time. Finally, it should not be neglected that thyroid hormones circulate bound to plasma proteins, and their biological action is exerted by the fraction (0.02–0.1%) of unbound or “free” form (4). Hence, the direct fT4 assays may be inaccurate in patients with severe systemic illness or abnormalities of protein binding, so caution is needed when interpreting such tests in this setting (4).

Unlike other forms of subclinical hypothyroidism in childhood, Hashimoto AIT is characterized by an increased likelihood for progression to overt hypothyroidism (47). Long-term cardiovascular outcomes in pediatric patients with Hashimoto thyroiditis are largely unknown due to lack of longitudinal prospective studies. In addition, clinical studies aimed to address benefit of L-T4 administration on future cardiovascular health are required. Available data indicate that statins also have certain immunomodulatory properties (125). Hence, the impact on conventional dyslipidemia treatment on thyroid autoimmunity should be explored in the future.

## Conclusions

Available evidence suggests that AIT is associated with profound changes of lipid profile, which are driven not only by a decrease in thyroid hormones, but also by chronic inflammation and disturbed redox balance. In light of the current scientific data, novel biomarkers of altered lipoprotein metabolism might provide more complete information regarding lipid profile of Hashimoto AIT patients and subsequent cardiovascular risk. However, lack of reliable results in pediatric AIT patients urges the need for more comprehensive studies aimed to explore characteristics of dyslipidemia in children with Hashimoto AIT and implications for their future cardiovascular health.

## Author Contributions

All authors listed have made a substantial, direct and intellectual contribution to the work, and approved it for publication.

### Conflict of Interest

The authors declare that the research was conducted in the absence of any commercial or financial relationships that could be construed as a potential conflict of interest.
